# Is PPAR*γ* a Prospective Player in HIV-1-Associated Bone Disease?

**DOI:** 10.1155/2009/421376

**Published:** 2009-03-23

**Authors:** Eoin J. Cotter, Patrick W. Mallon, Peter P. Doran

**Affiliations:** ^1^Clinical Research Center, University College Dublin, Belfield, 4 Dublin, Ireland; ^2^School of Medicine & Medical Science, University College Dublin, Belfield, 4 Dublin, Ireland

## Abstract

Currently infection with the human immunodeficiency virus-1 (HIV-1) is in most instances a chronic disease that can be controlled by effective antiretroviral therapy (ART). However, chronic use of ART has been associated with a number of toxicities; including significant reductions in bone mineral density (BMD) and disorders of the fat metabolism. The peroxisome proliferator-activated receptor gamma (PPAR*γ*) transcription factor is vital for the development and maintenance of mature and developing adipocytes. Alterations in PPAR*γ* expression have been implicated as a factor in the mechanism of HIV-1-associated lipodystrophy. Both reduced BMD and lipodystrophy have been well described as complications of HIV-1 infection and treatment, and a question remains as to their interdependence. Interestingly, both adipocytes and osteoblasts are derived from a common precursor cell type; the mesenchymal stem cell. The possibility that dysregulation of PPAR*γ* (and the subsequent effect on both osteoblastogenesis and adipogenesis) is a contributory factor in the lipid- and bone-abnormalities observed in HIV-1 infection and treatment has also been investigated. This review deals with the hypothesis that dysregulation of PPAR*γ* may underpin the bone abnormalities associated with HIV-1 infection, and treats the current knowledge and prospective developments, in our understanding of PPAR*γ* involvement in HIV-1-associated bone disease.

## 1. Introduction

Aside from the serious effects on the cells of the immune system, HIV-1 infection and its treatment have been associated with disorders in other tissues, most notably bone [1, 2] and adipose [3–6] tissues, where reduced bone mineral density (BMD) and abnormalities of the lipid metabolism (lipodystrophy, dyslipidemia, and insulin resistance) have been described. In both disorders (particularly those of the adipose tissue), antiretroviral treatment is believed to play a major role, but the contribution of underlying HIV-1 infection has yet to be elucidated, and therefore cannot be ignored as a potential causative factor.

**Figure 1:**
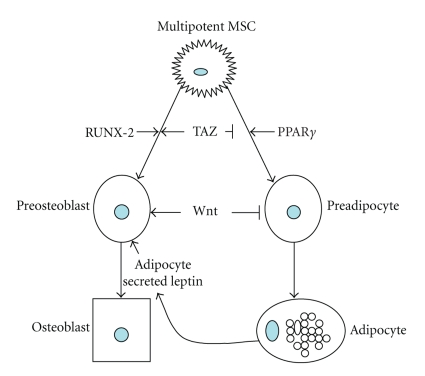
Factors governing normal osteogenesis and adipogenesis from mesenchymal stem cells. Multipotent mesenchymal stem cells can differentiate into a number of cell types, including adipocytes and osteoblasts. (⊥ indicates inhibition; ↓ indicates stimulation). The transcriptional coactivator Taz negatively regulates adipogenesis and promotes osteogenesis through suppression of PPAR*γ* and activation of RUNX-2, while overexpression of PPAR*γ* can reduce bone formation. Also, a number of other factors such as secreted proteins from Wnt family promote the differentiation and maintenance of osteoblasts while reducing the differentiation of the adipocytes. In addition, factors secreted by mature adipocytes, such as leptin and estrogen, can increase bone mass in vivo.

PPAR*γ* is a nuclear membrane bound transcription factor which regulates a number of genes involved in adipogenesis from common precursor cells type (mesenchymal stem cells), maturation of preadipocytes, lipid accumulation, and maintenance of adipogenic phenotype [7, 8]. As such, it is not surprising that a number of recent studies have indicated that certain drugs known to be associated with lipodystrophy dysregulate PPAR*γ* [9, 10]. The involvement of PPAR*γ* in HIV-1-associated bone disease is an area that has been little studied to date; however numerous studies suggest that PPAR*γ* plays a role in conditions such as osteoporosis in the absence of HIV-1 or ART, and increased adipocyte content of osteoporotic bone has been reported [10–12]. In addition, osteoblasts—the cells responsible for depositing bone—are derived from mesenchymal stem cells, and evidence suggests that the balance of PPAR*γ* and the pro-osteogenic runt-related transcription factor-2 (RUNX-2) is a key in the determination of mesenchymal stem cell fate [13–15] (see Figure 1). This review will introduce the current knowledge of the role of PPAR*γ* in bone biology in normal and disease states, and discuss its potential as a mechanism for HIV-1-associated bone disease.

## 2. HIV-1-Associated Bone Disease

Osteoporosis is defined as a reduction in the bone mass and disruption of the microarchitecture of the bone which leads to a greatly increased risk of fractures, while osteopenia is a lesser reduction in bone density and strength which may remain asymptomatic, but can precede actual osteoporosis. The world health organization (WHO) definitions specify t-scores between −1 and −2.5 as being indicative of osteopenia, while t-scores of less than −2.5 are indicative of osteoporosis [16]. Fractures resulting from osteoporosis affect one in two women and one in five men over the age of 50, and are a significant financial burden to health services, with an estimated combined annual cost of 30 billion Euro in the EU [17].

As will be discussed further, bone remodeling is dependent on the opposing functions of two cell types, osteoblasts, which make new bone (bone formation), and osteoclasts, which destroy old bone (bone resorption). Therefore, the balance between the number and activity of osteoclasts and osteoblasts is crucial in normal bone homeostasis; the perturbation of which can directly lead to increased bone fragility and fracture risk. Two important molecules: macrophage colony-stimulating factor (M-CSF) and receptor for activation of nuclear factor-kappa B ligand (RANKL) produced from osteoblast/stromal cells regulate the differentiation, function, and survival of osteoclasts, while the transcription factors, RUNX-2 and Osterix, have been reported to regulate osteoblast differentiation [18].

### 2.1. HIV-1 Infection and Bone Disease

Bone metabolism in HIV-infected individuals has been studied since the late 1980s, although the number of early studies is somewhat limited. Before the widespread use of highly active ART, studies indicated that bone mineral metabolism was only minimally affected in HIV-infected patients. Serrano et al. assessed histomorphometry in HIV-positive patients and found that parameters of histomorphometry such as serum osteocalcin were found to be lower in patients who, according to the Centers for Disease Control (CDC) classification, had greater disease severity [19]. Paton et al. reported that 45 HIV-infected patients had marginally lower BMD at the lumbar spine. None of the patients had reduced BMD to levels associated with a diagnosis of osteoporosis [20]. More recently however, it became clear that reduced BMD is also frequent in the absence of therapy [21–24]. In a study by McGowan et al., the prevalence of osteopenia among antiretroviral-naive HIV-positive individuals to be approximately 28%, which is approximately 50% greater than the expected incidence in the general, uninfected population [25]. Studies which have included patients with more advanced HIV disease who have received treatment for longer periods have reported prevalence of 40% to 50% [26, 27], placing reduced BMD among the most common HIV-1-associated metabolic toxicities. Amiel et al. also assessed BMD in 48 HIV-infected treatment-naive patients, 49 HIV-infected patients on protein inhibitors, 51 HIV-infected patients on no-protein inhibitors, and 81 HIV-uninfected control subjects. The results showed a significant decrease of BMD (9%) in all HIV-infected patients compared to the control subjects, occurring concurrently with a lower bone alkaline phosphatase and higher urinary cross-laps/Cr. [28]. The clinical impact of this reduced BMD is beginning to be examined; recent studies in a large American health care system, involving 8526 HIV infected patients and over 2 million control subjects, demonstrated that the prevalence of any fracture type was significantly higher in the HIV-infected population (2.87 versus 1.77 fractures/100 persons, *P* = .002). This study did not specify the treatment status of their subjects, but the data suggests that HIV-1-related fractures are a significant and growing clinical issue [29].

### 2.2. Antiviral Treatment and Bone Diseases

Antiretroviral treatment (ART) is a complex therapeutic regimen, in which patients typically take 2-3 agents selected from an array of 30 approved antiretroviral agents. ART, in general, comprises of two major therapeutic strategies: a protease inhibitor- (PI-) based regimen and a nucleoside reverse transcriptase inhibitor- (NRTI-) based regimen. The PI-based regimen uses one or two PIs combined with two NRTIs, whereas the NRTI-based regimen uses two NRTIs combined with one non-nucleoside reverse transcriptase inhibitor (NNRTI). With more effective therapies as a result of HAART, the prevalence of HAART-associated bone diseases has increased [30].

A higher incidence of reduced BMD has been clinically associated with both PI and NRTI uses. Tebas et al. determined that in HIV-1 patients receiving PIs about 50% of the patients had osteopenia and other 21% had osteoporosis [31]. This incidence is significantly increased compared to patients without therapy or normal controls. Studies by Moore et al. confirmed that 71% of HIV-infected patients on PI therapy have reduced BMD [32]. Similarly, Carr et al. reported that 3% of 44 HIV-infected patients receiving NRTIs developed osteoporosis and 22% developed osteopenia [33], while in a study examining HIV-1-infected men Mallon determined a reduction in BMD beginning at 48 weeks postinitiation of treatment [6]. Tsekes et al. determined BMD and whole body fat by dual energy X-ray absorbance (DEXA) of HIV-infected patients receiving zidovudine and other NRTIs and found significant decreases in both body fat and BMD [34]. In addition, the recent analysis by Brown and Qaqish [35] also reported 2.5-fold increased odds of reduced BMD in ART-treated patients compared with ART-naive patients (95% CI 1.8, 3.7). However, most studies are in agreement that traditional risk factors for osteoporosis, such as ethnic variations, female sex, increasing age, low body mass index, and time since menopause, are all independent predictors of osteopenia/osteoporosis [36–40].

In addition, it has been noted that HIV-infected patients have an increased risk for osteonecrosis of the hip [41]. Keruly et al. reported 15 cases of avascular hip necrosis in HIV-infected patients and suggested that the incidence of osteonecrosis in HIV-infected patients was higher than the general HIV-negative population [42]. It is not known whether this phenomenon is attributable to HIV-1 infection itself, HAART, or other HIV-associated complications.

The mechanisms by which either HIV-1 or its treatment induces reduced BMD are as yet unclear, and several researchers have suggested that reduced vitamin D levels observed in HIV-1-infected patients, and particularly the reduced levels of the biologically active metabolite 1,25(OH)_2_D (which is the natural ligand for the vitamin D receptor (VDR)), may contribute to reduced BMD [43]. Studies have demonstrated that the level of 1,25(OH)_2_D in HIV-1-infected patients is between 5 and 50% lower than that in infected patients [24, 44, 45]. In addition, studies have indicated that patients receiving treatment are more likely to have greater reductions in 1,25(OH)_2_D, with a recent Dutch study suggesting that NNRTI treatment may increase the risk of vitamin D deficiency [46, 47]. In addition, the latter study demonstrated that patients receiving treatment also have increased parathyroid hormone (PTH) levels, increasing the potential risk of reduced bone mass.

In short, HIV-1-associated bone disorders are a significant and increasingly well-defined clinical issue. However, the molecular basis underpinning these clinical observations remains to be fully explained.

## 3. PPAR*γ*: Mediator of Development and Disease in Bone Biology

As discussed previously, maintenance of bone homeostasis is mediated through a balance of osteoblast-mediated bone deposition and osteoclast-mediated bone resorption. The continued production of these cells from stromal (mesenchymal) and hematopoietic (monocyte) precursors, respectively, is an essential component in the maintenance of BMD. Stromal progenitor or mesencymal stem cells are multipotent cells, capable of producing cells of a number of different lineages, including osteoblasts and adipocytes [47–49].

Since the early 1990s, researchers have hypothesized that a “see-saw” relationship exists in the bone marrow cavity, where production of adipocytes from stromal precursors is at the expense of osteoblast production and vice versa [50, 51]. This theory is born out by a clinically observed phenomenon, such as the increased adipocyte content of osteoporotic and aging bone [51–53] as well as studies where agents inducing adipocyte production reduced osteoblast number [49, 50]. Likewise, treatment of bone marrow stromal cells with bone morphogenic proteins (BMPs) resulted in reduced formation of adipocytes [53]. Adipocytes can also produce secreted factors such leptin and estrogen, which can positively regulate bone mass [13, 54, 55], further underlining the interrelated nature of bone and fat development (see Figure 1).

PPARs are ligand-activated nuclear hormone receptors which stimulate expression of genes containing peroxisome proliferator response elements (PPREs) [53, 54]. There are three principal members of this family, PPAR*α*, PPAR*δ*, and PPAR*γ*, activation of which stimulates genes involved in fatty acid oxidation, uncoupling of respiration toward heat production (thermoregulation) and terminal adipocyte differentiation (including intracellular lipid accumulation), respectively, (see Table 1) [49, 50, 55–59].

The activity of PPAR*γ* and RUNX-2 is a key to our understanding of the relationship between fat and bone. Activity of the RUNX-2 transcription factor is not only essential for maintenance of osteoblast phenotype, but it is also involved in driving the differentiation of osteoblasts from mesenchymal stem cells [11–14], while activity of PPAR*γ* in mesenchymal stem cells induces differentiation into adipocytes. The eventual phenotype of the differentiating cell is generally considered to be controlled by an antagonistic balance between RUNX-2 and PPAR*γ* [13, 14]. Studies have demonstrated, for example, that activation of PPAR*γ* using pharmacological agents can lead to decreased bone mass in vivo, while mice lacking the PPAR*γ* gene display increased bone mass and an inability to develop adipocytes [59–61]. Indeed, even in the eventual mature cell, the function can be altered by dysregulating this balance, with in vivo studies using a mouse model demonstrating reduced bone formation rate and suppression of RUNX-2 in osteoblasts in which PPAR*γ* had been activated [61], while Kim et al. have demonstrated that activation of PPAR*γ* induces death through a MAPK-dependant mechanism in osteoblastic cells [62].

PPAR*γ* deficient mice (having a mutation in the PPAR*γ*2 locus) have been generated and display a “lipodystrophic” phenotype, which occurs concurrently with increased bone mass, to the point where the bone marrow is almost completely occluded and hematopoiesis moves to extramedullary sites, such as the spleen [61, 63]. Recently, our understanding of the roles of PPAR*γ* in numerous physiologic processes, including the bone/fat paradigm, has been furthered by the development of the thiazolidinedione (TDZ) family of PPAR*γ* ligands, such as netoglitazone, pioglitazone, rosiglitazone, and GW0072 [64–67]. Studies have demonstrated that treatment of murine osteoblasts with netoglitazone and GW0072 can block osteoblast differentiation, without inducing adipogenesis [62, 64], while in vivo studies have demonstrated that rosiglitazone, a ligand with higher affinity for PPAR*γ*, decreased bone mineral density, bone formation rate, and trabecular bone volume, while increasing adipogenesis [65, 67]. Further studies on ovariectomized rats revealed that these effects are mediated in part by the suppression of the RUNX-2 transcription factor [67], giving further strength to the argument that an antagonistic relationship between PPAR*γ* and RUNX-2 governs bone and fat formation. Indeed, Hong et al. have demonstrated that shared coactivator protein, TAZ, accounts in some part for this relationship, in that it coactivates RUNX-2 and bone formation, while suppressing PPAR*γ* [68].

### 3.1. PPAR*γ* in HIV-1-Associated Lipodystrophy

ART is associated with changes in fat metabolism, broadly termed lipodystrophy (changes in fat distribution) or lipoatrophy (atrophy of adipose tissue). Severe forms of lipodystrophy are a major cosmetic concern, and can lead to suboptimal adherence to therapy. In addition, lipodystrophy is associated with markers of cardiovascular risk, such as insulin resistance and dyslipidemia [5].

In vitro, expression of PPAR*γ* is decreased by exposure to anti-HIV-1 PI and NRTI drugs. In differentiating adipocytes, exposure to nelfinavir, saquinavir, and ritonavir at 10 *μ*M concentrations resulted in decreased adipogenesis and expression of the PPAR*γ*-mediated mRNA encoding aP2 and lipoprotein lipase (LPL) [10]. Similar effects on PPAR*γ* expression were observed in 3T3-F442A adipocyte cells exposed to 10–50 *μ*M indinavir [69], while studies by the same group have also demonstrated that the nuclear association of the PPAR*γ* regulator SREBP-1 is reduced by treatment with indinavir [70]. In mature adipocytes, inhibition of PPAR*γ* function by expression of a dominant negative PPAR*γ* isoform results in decreased accumulation of intracellular triglyceride, decreased cell size, and decreased expression of genes involved in both fatty acid and glucose metabolism, including the glucose transporter GLUT-4 [71]. In lipoatrophic mice, ablation of PPAR*γ* activity in liver resulted in hepatic steatosis, hypertriglyceridemia, and muscle insulin resistance [72]. Many of these features are shared by PI-treated patients with HIV-1-associated lipodystrophy.

In vivo, patients with lipodystrophy had lower adipose tissue expression of both PPAR*δ* and PPAR*γ* than those without lipodystrophy. This was accompanied by decreases in a number of PPAR*γ*-responsive downstream genes including LPL and GLUT-4 [73, 74]. In studies by Mallon, NRTI treatment of non-HIV-1-infected subjects (either stavudine/lamivudine or zidovudine/lamivudine for six weeks) resulted in reduced PPAR*γ* expression in adipose tissue (alongside alterations in transcription of mitochondrial DNA, and upregulation of genes associated with mitochondrial transcriptional regulation), although in this study the effects on overall fat mass were not determined [9].

In patients with type 2 diabetes, exposure to TZD, which act as PPAR*γ* ligands, resulted in increased expression of PPAR*γ*-target genes such as LPL and fatty acid synthase (FAS) in subcutaneous adipose tissue biopsies, without increasing expression of PPAR*γ* itself [71]. However, studies utilizing TZD to treat lipodystrophy have produced variable, and at best, modest results [75–78]. More recently, van Wijk et al. demonstrated that rosiglitazone treatment, compared to treatment with metformin, increased subcutaneous abdominal and visceral abdominal fat in lipodystrophy, however this was a small study (*n* = 39), was not blinded or placebo controlled, and did not measure clinical outcomes [79].

The weight clinical and scientific evidence suggests that HIV-1/ART-associated lipid abnormalities occur largely as a result of treatment rather than infection. However, a recent study raised the possibility that there may also be a viral component; Shrivastav et al. [80] demonstrated that treatment with the HIV-1 accessory viral protein R (Vpr) could suppress PPAR*γ*-induced transactivation in 3T3-L1 murine adipocyte cells, with a consequent inhibition of adipocyte differentiation. Vpr is a 96-amino-acid accessory protein, which is packaged in the viral capsid, and is found in the nucleus early after cell infection [81, 82]. Among the functions of Vpr is its ability to act as a transcriptional activator of viral and cellular promoters [83–86]. Vpr enhances the activity of steroid hormone receptors, including the gluticorticoid receptor (GR), which Vpr can bind via its LXXLL motif [85]. Studies involving cotransfection with constructs expressing wild type and mutant (LXXLL null) Vpr constructs with reporter constructs containing the PPRE demonstrated that this phenomenon was dependent on the LXXLL motif. Further experiments demonstrated that the GR did not play a role, and that Vpr and PPAR*γ* interacted directly in living cells. The authors of this study hypothesize that in vivo circulating Vpr, or Vpr produced as a result of direct infection of adipocytes, could suppress differentiation of preadipocytes in a PPAR*γ* dependent manner with obvious consequences for the development of lipodystrophy and insulin resistance [80].

### 3.2. PPAR*γ* in HIV-1-Associated Bone Disease

In contrast to the clearly defined role for PPAR*γ* in HIV-1/ART-associated lipid abnormalities, few studies have focused on its potential impact in HIV-1/ART-associated bone abnormalities.

To date, studies into mechanism of reduced bone density have been understandably focused on two distinct strands, namely, the effects on osteoblast and osteoclast number and function. In the case of OC research, several studies have demonstrated that osteoclast function can be altered in vitro by treatment with both ritonavir and HIV-1 gp120 [87, 88]. Jain et al. demonstrated that osteoclast activity, measured using a rat neonatal calvaria assay, increased in the presence of nelfinavir, indinavir, saquinavir, or ritonavir, while lopinavir and amprenavir did not increase osteoclast activity. In addition, Pan et al. reported a significant increase in markers of osteoclastogenesis (namely, the activity of the tartaric acid phosphatase (TRAP) promoter and the NF-*κ*b transcription factor) in RAW264.7 (mouse leukemic monocyte macrophage cell line cells) and primary mouse osteoclast precursors treated with the NRTI zidovudine [89]. This same group has more recently reported that the NRTIs ddi and lamiduvine also induced osteoclastogenesis in vitro and osteopenia in an in vivo mouse model [90].

Similarly, osteoblast-based studies have produced some interesting data. Clinically, Serrano et al. reported reduced numbers of osteoclasts in HIV patients; a phenomenon occurring along side-reduced serum osteocalcin levels and bone formation rate [19]. Previous and ongoing in vitro studies by our own group have demonstrated that osteoblast activity (as measured by calcium deposition and alkaline phosphatase activity) can be reduced by a number of antiretroviral drugs (including both nelfinavir and indinavir). In addition, these studies identified tissue inhibitor of metalloproteinase-3 (TIMP-3) as a mechanism for this observed loss in osteoblast function [91]. Further studies by our group demonstrated that treatment with the HIV-1 proteins p55-gag and gp120 reduced osteoblast activity in conjunction with reduction RUNX-2 transcription factor activity [92]. Interestingly, gp120 both decreased RUNX-2 activity and increased PPAR*γ*. Furthermore, our studies investigating the effect of HIV-1 proteins on mesenchymal stem cell differentiation have suggested that the proteins p55 and REV alter both mesenchymal stem cell osteoblastic differentiation and RUNX-2/PPAR*γ* signalling in nondifferentiating mesenchymal stem cells [93].

Although these studies used a somewhat simplistic model of HIV-1 exposure, given the evidence of the impact of PPAR*γ* on normal bone biology, and the observation that it can be perturbed in HIV-1-associated lipodystrophy, it is tempting to interpret these results as being suggestive of PPAR*γ* playing a role in HIV-1-mediated bone disease. However, there is an obvious stumbling block for this hypothesis, namely, that if increased PPAR*γ* activity in mesenchymal stem cell and osteoblasts could result in reduced bone mass, it would surely also increase fat mass. This picture is further complicated, as previously discussed studies have demonstrated that treatment of non-HIV-1-infected subjects with NNRTIs resulted in reduced PPAR*γ* expression in adipose tissue [9], while in vitro studies with 3T3-F442A cells have demonstrated that both PPAR*γ* expression and its association with SREBP-1 are reduced by treatment with indinavir [69, 70]. However, different processes may govern fat redistribution in different tissues, with gain in visceral fat and loss of subcutaneous fat. In addition, at least one ex vivo study suggests that both markers of adipocyte and osteoblastic differentiation are significantly reduced in human mesenchymal stem cells treated with a subset of protease inhibitors (particularly nelfinavir and saquinavir) [94], while HIV-1 patients receiving the NRTI zidovudine were shown to have reduced both BMD and whole body fat [33]. Could it be that contributing to both HIV-1/ART-associated bone and lipid disorders is an underlying disregulation of mesenchymal stem cell function combined with separate effects on adult or partially differentiated cells?

## 4. Conclusion

The importance of PPAR*γ* in both bone and fat metabolism has been clearly demonstrated, and while a role for PPAR*γ* in the lipid abnormalities associated with HIV-1 and its treatment is emerging, its involvement in HIV-1-associated bone disease remains unclear. Given the common origin of both adipocytes and osteoblasts from mesenchymal stem cell, and the demonstrated effect of increased PPAR*γ* expression on bone in vitro and in vivo, we hypothesize a potential role for PPAR*γ* in the reduced bone mass associated with HIV-1 infection and treatment. It may be possible that HIV-1 infection and/or treatment, through dysregulating PPAR*γ* (and possibly also RUNX-2) activity in undifferentiated stromal cells, or in partially differentiated preosteoblast and preadipocyte cells, can reduce the eventual number or functional capacity of the adult cell types.

In order to further investigate this hypothesis, it may be worthwhile to conduct ex vivo experiment on primary mesenchymal stem cells collected from HIV-1 patients. The expression and activity of PPAR*γ* and differentiation potential of these cells could be assessed and compared to those of cells harvested from uninfected individuals, and the data gathered used to generate a new model of HIV-1/PPAR*γ*/mesenchymal stem cell interactions.

It is clear that further studies are necessary to more fully describe the role of PPAR*γ* in the setting of HIV-1-associated bone disease and its interplay with vascular and fat disorders.

## Figures and Tables

**Table 1:** PPAR*γ*-regulated genes involved in adiogenesis, glucose uptake, and thermoregulation (↑ positive regulation; ↓ negative regulation).

PPAR*γ*-regulated genes		
Gene	Tissue/cell type	Function

CCAT enhancer binding protein *α* (CEBP*α*) [7]↑	Adipose/preadipose tissue	Transcription factor. CDK2/4 inhibition-cell cycle arrest
Adipose differentiation related protein (ADRP) [50]↑	Adipose/preadipose tissue	Associated with globule membrane, early marker of adipocyte differentiation
Lipoprotein lipase [49]↑	Vascular endothelium, heart, muscle, adipose	Lipid hydrolysis from lipoproteins
Adiponectin [49]↑	Adipose tissue (secreted)	Fatty acid catabolism
Adipocyte protein 2 (aP2/FABP4) [49]↑	Adipocytes/macrophages	Intercellular lipid transport
Tumour suppressor candidate 5 (TUSC 5) [56]↑	Preadipose/adipose tissue	Associated with entry into the later stages of adipogenesis
Glucose transporters 4 (GLUT) 4 [57]↑	Wide tissue distribution	Insulin stimulated glucose uptake
Uncoupling proteins 1-3 (UCP 1-3) [58]↑	Adipose tissue, skeletal muscle, liver	Thermogenesis/thermoregulation

## References

[1] Powderly WG (2002). Long-term exposure to lifelong therapies. *Journal of Acquired Immune Deficiency Syndromes*.

[2] Chew NS, Doran PP, Powderly WG (2007). Osteopenia and osteoporosis in HIV: pathogenesis and treatment. *Current Opinion in HIV and AIDS*.

[3] Milinkovic A, Martinez E (2005). Current perspectives on HIV-associated lipodystrophy syndrome. *Journal of Antimicrobial Chemotherapy*.

[4] Fisher K (2001). Wasting and lipodystrophy in patients infected with HIV: a practical approach in clinical practice. *AIDS Reader*.

[5] Milinković A (2006). HIV-associated lipodystrophy syndrome. *Collegium Antropologicum*.

[6] Mallon PW, Miller J, Cooper DA, Carr A (2003). Prospective evaluation of the effects of antiretroviral therapy on body composition in HIV-1-infected men starting therapy. *AIDS*.

[7] Rosen ED, Spiegelman BM (2000). Molecular regulation of adipogenesis. *Annual Review of Cell and Developmental Biology*.

[8] Heikkinen S, Auwerx J, Argmann CA (2007). PPAR*γ* in human and mouse physiology. *Biochimica et Biophysica Acta*.

[9] Mallon PW, Unemori P, Sedwell R (2005). In vivo, nucleoside reverse-transcriptase inhibitors alter expression of both mitochondrial and lipid metabolism genes in the absence of depletion of mitochondrial DNA. *Journal of Infectious Diseases*.

[10] Lenhard JM, Furfine ES, Jain RG (2000). HIV protease inhibitors block adipogenesis and increase lipolysis in vitro. *Antiviral Research*.

[11] Gimble JM, Zvonic S, Floyd ZE, Kassem M, Nuttall ME (2006). Playing with bone and fat. *Journal of Cellular Biochemistry*.

[12] Zhao L-J, Jiang H, Papasian CJ (2008). Correlation of obesity and osteoporosis: effect of fat mass on the determination of osteoporosis. *Journal of Bone and Mineral Research*.

[13] Shi X, Hamrick M, Isales CM (2007). Energy balance, myostatin, and GILZ: factors regulating adipocyte differentiation in belly and bone. *PPAR Research*.

[14] Caplan AI (1991). Mesenchymal stem cells. *Journal of Orthopaedic Research*.

[15] Gregory CA, Prockop DJ, Spees JL (2005). Non-hematopoietic bone marrow stem cells: molecular control of expansion and differentiation. *Experimental Cell Research*.

[16] Poole KES, Compston JE (2006). Osteoporosis and its management. *British Medical Journal*.

[17] NIH Consensus Development Panel (2001). Osteoporosis prevention, diagnosis, and therapy. *Journal of the American Medical Association*.

[18] Teitelbaum SL (2000). Bone resorption by osteoclasts. *Science*.

[19] Serrano S, Mariñoso ML, Soriano JC (1995). Bone remodelling in human immunodeficiency virus-1-infected patients. A histomorphometric study. *Bone*.

[20] Paton NIJ, Macallan DC, Griffin GE, Pazianas M (1997). Bone mineral density in patients with human immunodeficiency virus infection. *Calcified Tissue International*.

[21] Mondy K, Powderly WG, Claxton SA (2005). Alendronate, vitamin D, and calcium for the treatment of osteopenia/osteoporosis associated with HIV infection. *Journal of Acquired Immune Deficiency Syndromes*.

[22] Knobel H, Guelar A, Vallecillo G, Nogués X, Díez A (2001). Osteopenia in HIV-infected patients: is it the disease or is it the treatment?. *AIDS*.

[23] Lawal A, Engelson ES, Wang J, Heymsfield SB, Kotler DP (2001). Equivalent osteopenia in HIV-infected individuals studied before and during the era of highly active antiretroviral therapy. *AIDS*.

[24] Teichmann J, Stephan E, Lange U (2003). Osteopenia in HIV-infected women prior to highly active antiretroviral therapy. *Journal of Infection*.

[25] McGowan I, Cheng A, Coleman S, Johnson A, Genant H Assessment of bone mineral density (BMD) in HIV-infected antiretroviral-therapy-naive patients.

[26] Hoy J, Hudson J, Law M, Cooper DA Osteopenia in a randomized, multicenter study of protease inhibitor (PI) substitution in patients with the lipodystrophy syndrome and well-controlled HIV viremia.

[27] Mondy K, Yarasheski K, Powderly WG (2003). Longitudinal evolution of bone mineral density and bone markers in human immunodeficiency virus-infected individuals. *Clinical Infectious Diseases*.

[28] Amiel C, Ostertag A, Slama L (2004). BMD is reduced in HIV-infected men irrespective of treatment. *Journal of Bone and Mineral Research*.

[29] Triant VA, Brown TT, Lee H, Grinspoon SK (2008). Fracture prevalence among human immunodeficiency virus (HIV)-infected *versus* non-HIV-infected patients in a large U.S. healthcare system. *The Journal of Clinical Endocrinology & Metabolism*.

[30] Pan G, Yang Z, Ballinger SW, McDonald JM (2006). Pathogenesis of osteopenia/osteoporosis induced by highly active anti-retroviral therapy for AIDS. *Annals of the New York Academy of Sciences*.

[31] Tebas P, Powderly WG, Claxton S (2000). Accelerated bone mineral loss in HIV-infected patients receiving potent antiretroviral therapy. *AIDS*.

[32] Moore AL, Vashisht A, Sabin CA (2001). Reduced bone mineral density in HIV-positive individuals. *AIDS*.

[33] Carr A, Miller J, Eisman JA, Cooper DA (2001). Osteopenia in HIV-infected men: association with asymptomatic lactic acidemia and lower weight pre-antiretroviral therapy. *AIDS*.

[34] Tsekes G, Chrysos G, Douskas G (2002). Body composition changes in protease inhibitor-naive HIV-infected patients treated with two nucleoside reverse transcriptase inhibitors. *HIV Medicine*.

[35] Brown TT, Qaqish RB (2006). Antiretroviral therapy and the prevalence of osteopenia and osteoporosis: a meta-analytic review. *AIDS*.

[36] Yin M, Dobkin J, Brudney K (2005). Bone mass and mineral metabolism in HIV+ postmenopausal women. *Osteoporosis International*.

[37] Gallant JE, Dejesus E, Arribas JR (2006). Tenofovir DF, emtricitabine, and efavirenz vs. zidovudine, lamivudine, and efavirenz for HIV. *The New England Journal of Medicine*.

[38] Gallant JE, Staszewski S, Pozniak AL (2004). Efficacy and safety of tenofovir DF vs stavudine in combination therapy in antiretroviral-naive patients: a 3-year randomized trial. *Journal of the American Medical Association*.

[39] Dolan SE, Kanter JR, Grinspoon S (2006). Longitudinal analysis of bone density in human immunodeficiency virus-infected women. *The Journal of Clinical Endocrinology & Metabolism*.

[40] Wang MW-H, Wei S, Faccio R (2004). The HIV protease inhibitor ritonavir blocks osteoclastogenesis and function by impairing RANKL-induced signaling. *The Journal of Clinical Investigation*.

[41] Allison GT, Bostrom MP, Glesby MJ (2003). Osteonecrosis in HIV disease: epidemiology, etiologies, and clinical management. *AIDS*.

[42] Keruly JC, Chaisson RE, Moore RD (2001). Increasing incidence of avascular necrosis of the hip in HIV-infected patients. *Journal of Acquired Immune Deficiency Syndromes*.

[43] Villamor E (2006). A potential role for vitamin D on HIV infection?. *Nutrition Reviews*.

[44] Teichmann J, Stephan E, Discher T (2000). Changes in calciotropic hormones and biochemical markers of bone metabolism in patients with human immunodeficiency virus infection. *Metabolism*.

[45] Madeddu G, Spanu A, Solinas P (2004). Bone mass loss and vitamin D metabolism impairment in HIV patients receiving highly active antiretroviral therapy. *Quarterly Journal of Nuclear Medicine and Molecular Imaging*.

[46] Van Den Bout-Van Den Beukel CJP, Fievez L, Michels M (2008). Vitamin D deficiency among HIV type 1-infected individuals in the Netherlands: effects of antiretroviral therapy. *AIDS Research and Human Retroviruses*.

[47] Gregory CA, Prockop DJ, Spees JL (2005). Non-hematopoietic bone marrow stem cells: molecular control of expansion and differentiation. *Experimental Cell Research*.

[48] Nuttall ME, Gimble JM (2004). Controlling the balance between osteoblastogenesis and adipogenesis and the consequent therapeutic implications. *Current Opinion in Pharmacology*.

[49] Beresford JN, Bennett JH, Devlin C, Leboy PS, Owen ME (1992). Evidence for an inverse relationship between the differentiation of adipocytic and osteogenic cells in rat marrow stromal cell cultures. *Journal of Cell Science*.

[50] Dorheim M-A, Sullivan M, Dandapani V (1993). Osteoblastic gene expression during adipogenesis in hematopoietic supporting murine bone marrow stromal cells. *Journal of Cellular Physiology*.

[51] Verma S, Rajaratnam JH, Denton J, Hoyland JA, Byers RJ (2002). Adipocytic proportion of bone marrow is inversely related to bone formation in osteoporosis. *Journal of Clinical Pathology*.

[52] Meunier P, Aaron J, Edouard C, Vignon G (1971). Osteoporosis and the replacement of cell populations of the marrow by adipose tissue. A quantitative study of 84 iliac bone biopsies. *Clinical Orthopaedics and Related Research*.

[53] Gimble JM, Morgan C, Kelly K (1995). Bone morphogenetic proteins inhibit adipocyte differentiation by bone marrow stromal cells. *Journal of Cellular Biochemistry*.

[54] Hamrick MW (2007). Invited perspective: leptin and bone—a consensus emerging?. *Bonekey Osteovision*.

[55] Hamrick MW, Della-Fera MA, Choi Y-H, Pennington C, Hartzell D, Baile CA (2005). Leptin treatment induces loss of bone marrow adipocytes and increases bone formation in leptin-deficient *ob/ob* mice. *Journal of Bone and Mineral Research*.

[56] Oort PJ, Warden CH, Baumann TK, Knotts TA, Adams SH (2007). Characterization of Tusc5, an adipocyte gene co-expressed in peripheral neurons. *Molecular and Cellular Endocrinology*.

[57] Liao W, Nguyen MTA, Yoshizaki T (2007). Suppression of PPAR-*γ* attenuates insulin-stimulated glucose uptake by affecting both GLUT1 and GLUT4 in 3T3-L1 adipocytes. *American Journal of Physiology*.

[58] Kelly LJ, Vicario PP, Thompson GM (1998). Peroxisome proliferator-activated receptors *γ* and *α* mediate in vivo regulation of uncoupling protein (UCP-1, UCP-2, UCP-3) gene expression. *Endocrinology*.

[59] Ferré P (2004). The biology of peroxisome proliferator-activated receptors: relationship with lipid metabolism and insulin sensitivity. *Diabetes*.

[60] Evans RM, Barish GD, Wang Y-X (2004). PPARs and the complex journey to obesity. *Nature Medicine*.

[61] Akune T, Ohba S, Kamekura S (2004). PPAR*γ* insufficiency enhances osteogenesis through osteoblast formation from bone marrow progenitors. *The Journal of Clinical Investigation*.

[62] Kim SH, Yoo CI, Kim HT, Park JY, Kwon CH, Kim YK (2006). Activation of peroxisome proliferator-activated receptor-*γ* (PPAR*γ*) induces cell death through MAPK-dependent mechanism in osteoblastic cells. *Toxicology and Applied Pharmacology*.

[63] Cock T-A, Back J, Elefteriou F (2004). Enhanced bone formation in lipodystrophic PPAR*γ*
^*hyp*/*hyp*^ mice relocates haematopoiesis to the spleen. *EMBO Reports*.

[64] Lazarenko OP, Rzonca SO, Suva LJ, Lecka-Czernik B (2006). Netoglitazone is a PPAR-gamma ligand with selective effects on bone and fat. *Bone*.

[65] Ali AA, Weinstein RS, Stewart SA, Parfitt AM, Manolagas SC, Jilka RL (2005). Rosiglitazone causes bone loss in mice by suppressing osteoblast differentiation and bone formation. *Endocrinology*.

[66] Lecka-Czernik B, Moerman EJ, Grant DF, Lehmann JM, Manolagas SC, Jilka RL (2002). Divergent effects of selective peroxisome proliferator-activated receptor-*γ*2 ligands on adipocyte *versus* osteoblast differentiation. *Endocrinology*.

[67] Rzonca SO, Suva LJ, Gaddy D, Montague DC, Lecka-Czernik B (2004). Bone is a target for the antidiabetic compound rosiglitazone. *Endocrinology*.

[68] Hong J-H, Hwang ES, McManus MT (2005). TAZ, a transcriptional modulator of mesenchymal stem cell differentiation. *Science*.

[69] Caron M, Auclair M, Vigouroux C, Glorian M, Forest C, Capeau J (2001). The HIV protease inhibitor indinavir impairs sterol regulatory element-binding protein-1 intranuclear localization, inhibits preadipocyte differentiation, and induces insulin resistance. *Diabetes*.

[70] Caron M, Auclair M, Sterlingot H, Kornprobst M, Capeau J (2003). Some HIV protease inhibitors alter lamin A/C maturation and stability, SREBP-1 nuclear localization and adipocyte differentiation. *AIDS*.

[71] Tamori Y, Masugi J, Nishino N, Kasuga M (2002). Role of peroxisome proliferator-activated receptor-*γ* in maintenance of the characteristics of mature 3T3-L1 adipocytes. *Diabetes*.

[72] Gavrilova O, Haluzik M, Matsusue K (2003). Liver peroxisome proliferator-activated receptor *γ* contributes to hepatic steatosis, triglyceride clearance, and regulation of body fat mass. *The Journal of Biological Chemistry*.

[73] Kannisto K, Sutinen J, Korsheninnikova E (2003). Expression of adipogenic transcription factors, peroxisome proliferator-activated receptor gamma co-activator 1, IL-6 and CD45 in subcutaneous adipose tissue in lipodystrophy associated with highly active antiretroviral therapy. *AIDS*.

[74] Bogacka I, Xie H, Bray GA, Smith SR (2004). The effect of pioglitazone on peroxisome proliferator-activated receptor-*γ* target genes related to lipid storage in vivo. *Diabetes Care*.

[75] Hadigan C, Yawetz S, Thomas A, Havers F, Sax PE, Grinspoon S (2004). Metabolic effects of rosiglitazone in HIV lipodystrophy: a randomized, controlled trial. *Annals of Internal Medicine*.

[76] Carr A, Workman C, Carey D (2004). No effect of rosiglitazone for treatment of HIV-1 lipoatrophy: randomised, double-blind, placebo-controlled trial. *The Lancet*.

[77] Sutinen J, Häkkinen A-M, Westerbacka J (2003). Rosiglitazone in the treatment of HAART-associated lipodystrophy—a randomized double-blind placebo-controlled study. *Antiviral Therapy*.

[78] Kamin D, Hadigan C, Lehrke M, Mazza S, Lazar MA, Grinspoon S (2005). Resistin levels in human immunodeficiency virus-infected patients with lipoatrophy decrease in response to rosiglitazone. *The Journal of Clinical Endocrinology & Metabolism*.

[79] van Wijk JPH, de Koning EJP, Cabezas MC (2005). Comparison of rosiglitazone and metformin for treating HIV lipodystrophy: a randomized trial. *Annals of Internal Medicine*.

[80] Shrivastav S, Kino T, Cunningham T (2008). Human Immunodeficiency Virus (HIV)-1 viral protein R suppresses transcriptional activity of peroxisome proliferator-activated receptor *γ* and inhibits adipocyte differentiation: implications for HIV-associated lipodystrophy. *Molecular Endocrinology*.

[81] Paxton W, Connor RI, Landau NR (1993). Incorporation of Vpr into human immunodeficiency virus type 1 virions: requirement for the p6 region of gag and mutational analysis. *Journal of Virology*.

[82] Heinzinger NK, Bukrinsky MI, Haggerty SA (1994). The Vpr protein of human immunodeficiency virus type 1 influences nuclear localization of viral nucleic acids in nondividing host cells. *Proceedings of the National Academy of Sciences of the United States of America*.

[83] He J, Choe S, Walker R, Di Marzio P, Morgan DO, Landau NR (1995). Human immunodeficiency virus type 1 viral protein R (Vpr) arrests cells in the G2 phase of the cell cycle by inhibiting p34(cdc2) activity. *Journal of Virology*.

[84] Jacotot E, Ferri KF, El Hamel C (2001). Control of mitochondrial membrane permeabilization by adenine nucleotide translocator interacting with HIV-1 viral protein R and Bcl-2. *The Journal of Experimental Medicine*.

[85] Sherman MP, De Noronha CMC, Pearce D, Greene WC (2000). Human immunodeficiency virus type 1 Vpr contains two leucine-rich helices that mediate glucocorticoid receptor coactivation independently of its effects on G_2_ cell cycle arrest. *Journal of Virology*.

[86] Kino T, Chrousos GP (2007). Virus-mediated modulation of the host endocrine signaling systems: clinical implications. *Trends in Endocrinology and Metabolism*.

[87] Wang MW-H, Wei S, Faccio R (2004). The HIV protease inhibitor ritonavir blocks osteoclastogenesis and function by impairing RANKL-induced signaling. *The Journal of Clinical Investigation*.

[88] Fakruddin JM, Laurence J (2003). HIV envelope gp120-mediated regulation of osteoclastogenesis via receptor activator of nuclear factor *κ*B ligand (RANKL) secretion and its modulation by certain HIV protease inhibitors through interferon-*γ*/RANKL cross-talk. *The Journal of Biological Chemistry*.

[89] Pan G, Wu X, McKenna MA, Feng X, Nagy TR, McDonald JM (2004). AZT enhances osteoclastogenesis and bone loss. *AIDS Research and Human Retroviruses*.

[90] Pan G, Kilby M, McDonald JM (2006). Modulation of osteoclastogenesis induced by nucleoside reverse transcriptase inhibitors. *AIDS Research and Human Retroviruses*.

[91] Malizia AP, Cotter EJ, Chew N, Powderly WG, Doran PP (2007). HIV protease inhibitors selectively induce gene expression alterations associated with reduced calcium deposition in primary human osteoblasts. *AIDS Research and Human Retroviruses*.

[92] Cotter EJ, Malizia AP, Chew N, Powderly WG, Doran PP (2007). HIV proteins regulate bone marker secretion and transcription factor activity in cultured human osteoblasts with consequent potential implications for osteoblast function and development. *AIDS Research and Human Retroviruses*.

[93] Cotter EJ, Ip HSM, Powderly WG, Doran PP (2008). Mechanism of HIV protein induced modulation of mesenchymal stem cell osteogenic differentiation. *BMC Musculoskeletal Disorders*.

[94] Jain RG, Lenhard JM (2002). Select HIV protease inhibitors alter bone and fat metabolism ex vivo. *The Journal of Biological Chemistry*.

